# Photobiomodulation of human dermal fibroblasts in vitro: decisive role of cell culture conditions and treatment protocols on experimental outcome

**DOI:** 10.1038/s41598-017-02802-0

**Published:** 2017-06-05

**Authors:** C. Mignon, N. E. Uzunbajakava, B. Raafs, N. V. Botchkareva, D. J. Tobin

**Affiliations:** 1 Philips Research, High Tech Campus 11, 5656 AE Eindhoven, The Netherlands; 2 Centre for Skin Sciences, Faculty of Life Sciences, University of Bradford, Richmond Road, BD7 1DP Bradford, West Yorkshire UK

## Abstract

Photobiomodulation-based (LLLT) therapies show tantalizing promise for treatment of skin diseases. Confidence in this approach is blighted however by lamentable inconsistency in published experimental designs, and so complicates interpretation. Here we interrogate the appropriateness of a range of previously-reported treatment parameters, including light wavelength, irradiance and radiant exposure, as well as cell culture conditions (e.g., serum concentration, cell confluency, medium refreshment, direct/indirect treatment, oxygen concentration, etc.), in primary cultures of normal human dermal fibroblasts exposed to visible and near infra-red (NIR) light. Apart from irradiance, all study parameters impacted significantly on fibroblast metabolic activity. Moreover, when cells were grown at atmospheric O_2_ levels (i.e. 20%) short wavelength light inhibited cell metabolism, while negligible effects were seen with long visible and NIR wavelength. By contrast, NIR stimulated cells when exposed to dermal tissue oxygen levels (approx. 2%). The impact of culture conditions was further seen when inhibitory effects of short wavelength light were reduced with increasing serum concentration and cell confluency. We conclude that a significant source of problematic interpretations in photobiomodulation reports derives from poor optimization of study design. Further development of this field using *in vitro*/*ex vivo* models should embrace significant standardization of study design, ideally within a design-of-experiment setting.

## Introduction

Photobiomodulation (PBM) or low-level light therapy (LLLT)^1^ is a rapidly expanding field, showing encouraging results for treatment of cutaneous disorders^2, 3^ and a wider range of health conditions^4–6^.

In dermatology, application of PBM spans the cosmetic and medical fields including androgenetic alopecia^7, 8^, alopecia areata^9^, skin rejuvenation^10^, wound healing^11^ and alleviation of psoriasis vulgaris and atopic dermatitis symptoms^12, 13^, where many commercialized professional and home-use devices exist^3, 14^.

Therapeutic action of PBM is attributed to non-thermal photochemical or photobiological action of light via interaction with a range of endogenous photoreceptors and chromophores contained in human skin^3, 15, 16^. Photoreceptors and mediators of the photobiological action of visible and NIR light include cytochrome c oxidase, nitrosated and flavo- proteins, opsins and ion-gated channels^3, 17–19^. Meanwhile, investigations on potential action mechanisms of PBM using *in vitro* and *ex vivo* models continue, but often reporting very contradictory results^2, 7, 20, 21^.

A remarkably wide range of optical parameters has been applied to both *in vitro* and *ex vivo* model systems (variable across 2 orders of magnitude), where negative, positive and neutral outcomes are reported for visible and near-infrared (NIR) light despite very similar optical settings^3, 8, 9, 11^. For example, exposure of human epidermal keratinocytes to similar doses of short wavelength visible light (i.e., 420 and 450 nm) were reported to alternately induce and reduce expression of differentiation markers^20, 22^.

Clarity on how one particular optical setting truly impacts on cell behavior is further compromised in published reports that don’t even disclose parameters applied or if they do sadly report them incorrectly^3^.

It now appears clear that gene expression patterns can be influenced in a dynamic time-varying manner by a single light treatment of defined parameters further suggesting that experimental outcomes are tightly coupled to treatment regime^23^.

Previously we have demonstrated that variations in treatment protocol and cell culture conditions result in different outcomes despite using the same light parameter^24^. In particular, interaction between components of cell culture medium and blue light (450 nm) can lead to cytotoxicity in human dermal fibroblasts when irradiated in the DMEM culture medium, but that this effect can be prevented by medium refreshment directly after light treatment^24^.

Therefore, we hypothesized that *in vitro* cell response to light is not exclusively defined by optical parameters and regimes, but rather also depends on treatment protocols. Variations in the latter may account for the myriad inconsistencies in the PBM literature^3^.

A number of factors can potentially affect PBM experimental outcomes such as cell confluency, passage, donor, donor age, body site, as well as differences in gene expression programs between primary cells and cell lines and many others even more difficult to apprehend such as the lack of standardization of the composition of bioactive compounds in FBS batch^25, 26^. Additional factors will also involve the biological diversity of any one histologically-distinct cell type e.g., different subtypes of skin dermal fibroblast (DF)^27, 28^ including the highly proliferative and low metabolically active papillary, and the less proliferative, more metabolically active reticular dermal fibroblasts^29^ and other cutaneous fibroblast subpopulations such as dermal sheath and dermal papilla fibroblasts of the hair follicle^29^ with strikingly different morphology, metabolism and gene expression. It is expected that these different cutaneous fibroblast subtypes will respond differently to light^30, 31^.

Therefore robust assessment of the therapeutic value of PBM requires multidimensional investigations, where optical (wavelength, radiant exposure, irradiance) and biological factors (cell type, subtype), as well as treatment protocols (environment of the cells during culture and treatment, supplements/components in growth medium, treatment iteration etc.) all need to be evaluated.

The aim of this study was to investigate the impact of several key factors such as wavelength, irradiance, radiant exposure, serum concentration, cell culture confluency, environmental oxygen concentration, light-based treatment regime and cell culture protocols on the response to light of human dermal fibroblasts *in vitro*.

## Results

### Impact of optical parameters on the response of human dermal fibroblasts to light treatment

**Figure 1:**
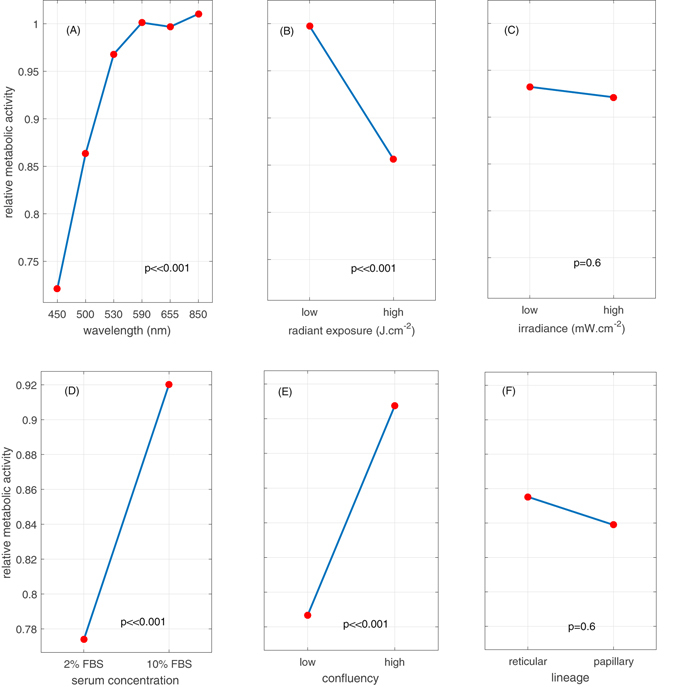
Main effects plot of the wavelength, radiant exposure, irradiance, serum concentration, initial cell confluency and lineage on the relative metabolic activity of fibroblasts. Main effects plot of the wavelength (**A**), radiant exposure (**B**) and irradiance (**C**) on the relative metabolic activity of human dermal fibroblasts (reticular and papillary). Data points correspond to grand means, i.e. the average value of the metabolic activity for each level of one selected factor (wavelength, dose or irradiance) while all the levels of the two other factors are averaged. Statistical significance was evaluated using ANOVA (N = 3, 2 lineages and 3 replicates). The levels of radiant exposure correspond to 2 J.cm^−2^ (low) and 30 J.cm^−2^ (high), the levels of irradiance depended on wavelength and are reported in Table 1 in the Materials and Methods Section. Main effects plot of serum concentration (**D**), initial confluency (**E**) and lineage (**F**) on the relative response of the metabolic activity of human dermal fibroblasts after light treatment (450 nm, 2 to 60 J.cm^−2^). Data points correspond to grand means, i.e. the average value of the metabolic activity for each level of one selected factor (serum concentration, confluency, lineage) while all the levels of the two other factors are averaged. Statistical significance was evaluated using ANOVA (N = 2, 2 lineages and 3 replicates). The initial ratio at the moment of seeding between ‘high’ and ‘low’ confluency groups is 5. The control group is has an average relative metabolic activity of 1.

Accordingly to a body of research on PBM, the wavelength of light and the radiant exposure exert the most profound effect on cell response to optical radiation, moreover reports indicating importance of irradiance in defining cell behavior upon irradiation can also be found in literature^2, 32^. Indeed, our results demonstrate that wavelength (p < 0.001) and radiant exposure (p < 0.001) of light had the strongest impact on the metabolic activity of human DF. At the same time, irradiance showed no or little effect (p = 0.6) (Fig. 1A–C). We observed that, on average, shorter wavelengths (450, 500 and 530 nm) have a large impact on fibroblast metabolic activity, while long wavelengths (>550 nm) show negligible effects within the tested radiant exposures when cells were cultured at 20% environmental oxygen (atmospheric level).

**Figure 2:**
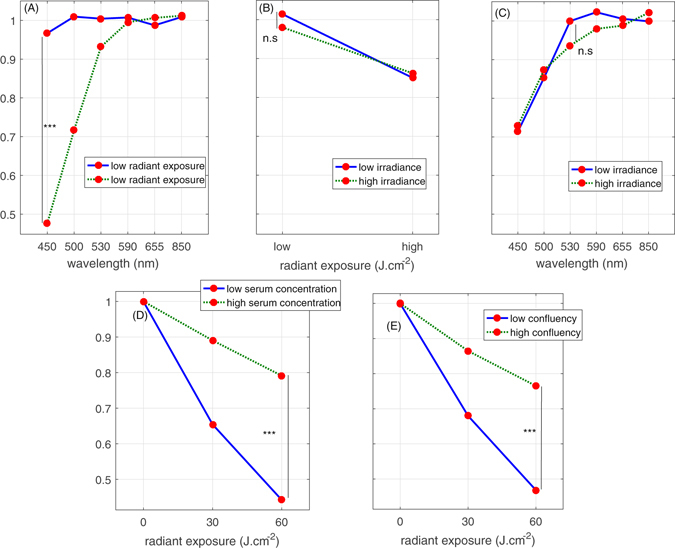
Interactions plot between the wavelength, radiant exposure, irradiance, cell confluency and serum concentration and their relative impact on human dermal fibroblasts metabolic activity. Interactions between wavelength and radiant exposure (**A**), irradiance and radiant exposure (**B**) and irradiance and wavelength (**C**) and their relative impact on human dermal fibroblasts metabolic activity (reticular and papillary). Data points correspond to grand means, i.e. the average value of the metabolic activity for each level of two selected factors (wavelength and radiant exposure, radiant exposure and irradiance or wavelength and irradiance) while all the levels of the remaining factor are averaged. Statistical significance was evaluated using ANOVA (N = 3, 2 lineages and 3 replicates). The levels of radiant exposure correspond to 2 J.cm^−2^ (low) and 30 J.cm^−2^ (high), the levels of irradiance depended on wavelength and are reported in Table 1 in the Materials and Methods Section. Interactions between levels of radiant exposure of 450 nm light and serum concentration (**D**), and initial confluency (**E**) and their impact on the relative response of the metabolic activity of human dermal fibroblasts. Data points correspond to grand means, i.e. the average value of the metabolic activity for each level of two selected factors (radiant exposure and serum concentration or radiant exposure and confluency) while all the levels of the remaining factor are averaged. Statistical significance was evaluated using ANOVA (N = 2, 2 lineages and 3 replicates). The initial ratio at the moment of seeing between ‘high’ and ‘low’ confluency groups is 5. The control group has an average relative metabolic activity of 1.

Wavelength and radiant exposure of light were strongly linked (Fig. 2A p < 0.001), whereby a radiant exposure-dependent cell behavior was seen with the short visible wavelengths (<550 nm). Low light exposure (2 J.cm^−2^) exhibited essentially neutral effects on fibroblast metabolic activity at all wavelengths, while cell inhibitory effects were seen at high exposures of light at 450, 500 and 530 nm (Fig. 2).

By contrast, no interaction was observed between irradiance levels and any of the optical factors (i.e. wavelength and radiant exposure, Fig. 2B,C) in terms of cell metabolic activity, specifically meaning that a choice of irradiance at a fixed dose and a wavelength did not impact cell response.

### Impact of biological factors on the response of human dermal fibroblasts to light treatment

To assess the impact of several biological factors on the response of DFs to light we selected a single wavelength (450 nm) as it exerted the strongest effect on the relative metabolic activity of the target cells, and varied radiant exposure from 2 J.cm^−2^ to high 60 J.cm^−2^ (Fig. 1D–F).

Fibroblast confluency and serum concentration strongly influence how specific light parameters affect cell behavior (Fig. 1D,E). In particular, lower cell confluency or lower serum concentration resulted in stronger light-associated inhibition of cell metabolic activity (Fig. 2D,E). Both lineages of fibroblasts (i.e., reticular and papillary) responded similarly to test light parameters (Fig. 1F).

**Figure 3:**
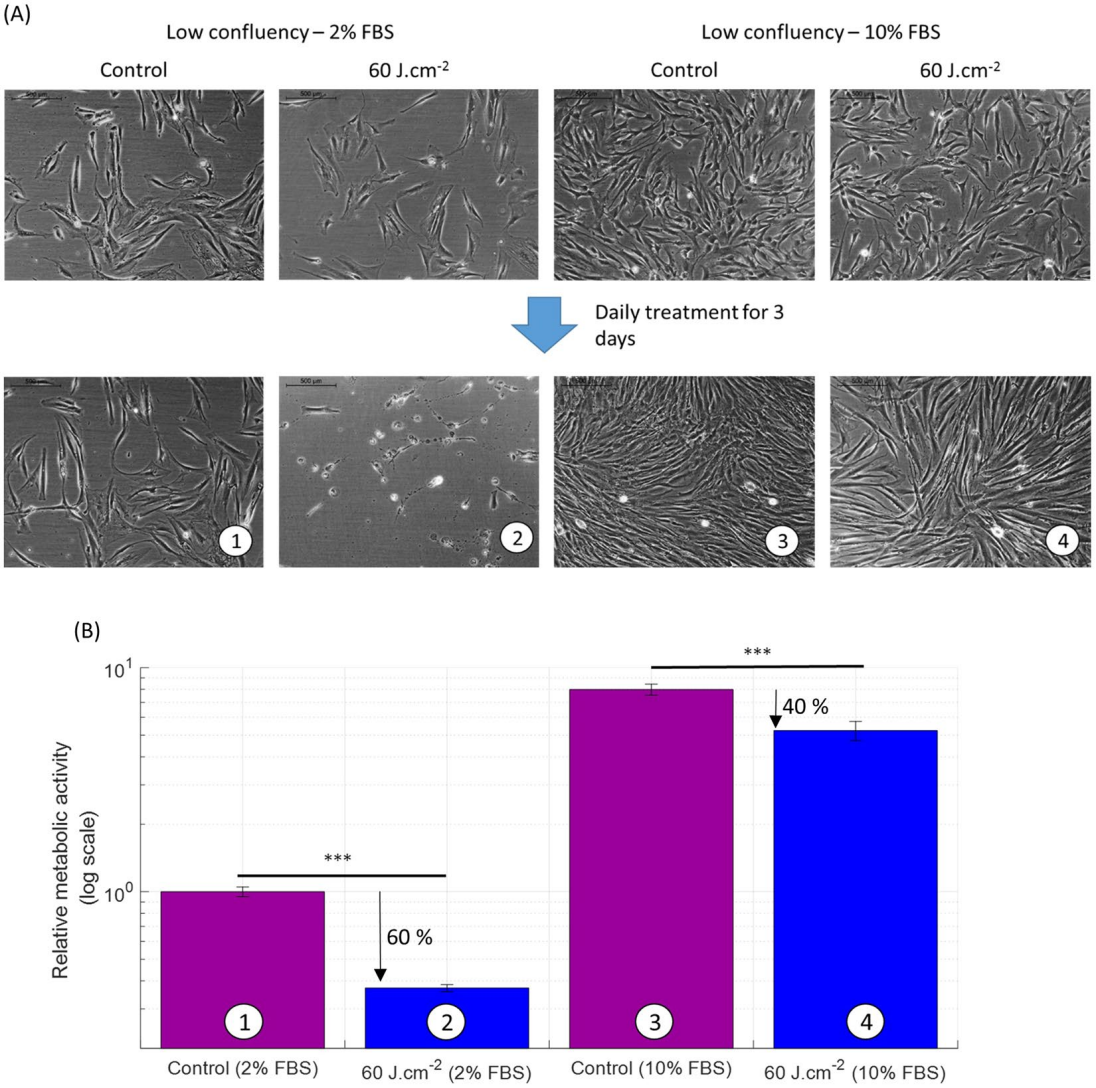
Serum concentration in the culture medium reduces the inhibitory effect of blue light (450 nm) on the metabolic activity of human dermal fibroblasts. (**A**) Phase-contrast images of human reticular fibroblasts on day 0 (8 days after seeding) at low initial confluency and cultured in DMEM with two serum concentrations 2% and 10% (upper line), and after three daily light treatments on day 4 at a low initial confluency (lower line). Papillary fibroblasts were found to behave similarly (not shown). A second donor responded similarly (not shown). (**B**) Relative metabolic activity of human reticular fibroblasts after light treatment at 450 nm, 50 mW.cm^−2^ with 60 J.cm^−2^ (log scale). We observe a 60% reduction in 2% FBS against 40% reduction in 10% FBS case compared to control. The high confluency level showed similar variation as a function of the change in FBS concentration (not shown), except for the cytotoxic effects observed in low serum/low confluency only. Statistical significance was evaluated using ANOVA. A second donor responded similarly (not shown as the scale is absolute). The control group has an average relative metabolic activity of 1. (N = 2, 2 lineages and 3 replicates).

As expected, serum concentration and confluency were related. High serum concentrations drove higher fibroblast proliferation and so confluency, given equal initial seeding density (Fig. 3, A B control bars).

In order to isolate the effect of serum concentration alone on the impact of light treatment, cells both at low and high confluency were treated using the same light parameter and protocol at the two culture conditions, 2% FBS and 10% FBS. At 60 J.cm^−2^ (Fig. 3B) the reduction of the metabolic activity of the fibroblasts grown in low serum was much stronger than at high serum, (60% versus 40%, respectively). This effect was also reflected in changes in cell morphology. Light treatment (450 nm, 60 J.cm^−2^) in low serum and low confluency fibroblasts triggered cytotoxic effects (Fig. 3A), while cells in high serum and low confluency (Fig. 3A) and in combinations of high confluency/low serum and high confluency/high serum (data not shown) exhibited no cytotoxic effects.

**Figure 4:**
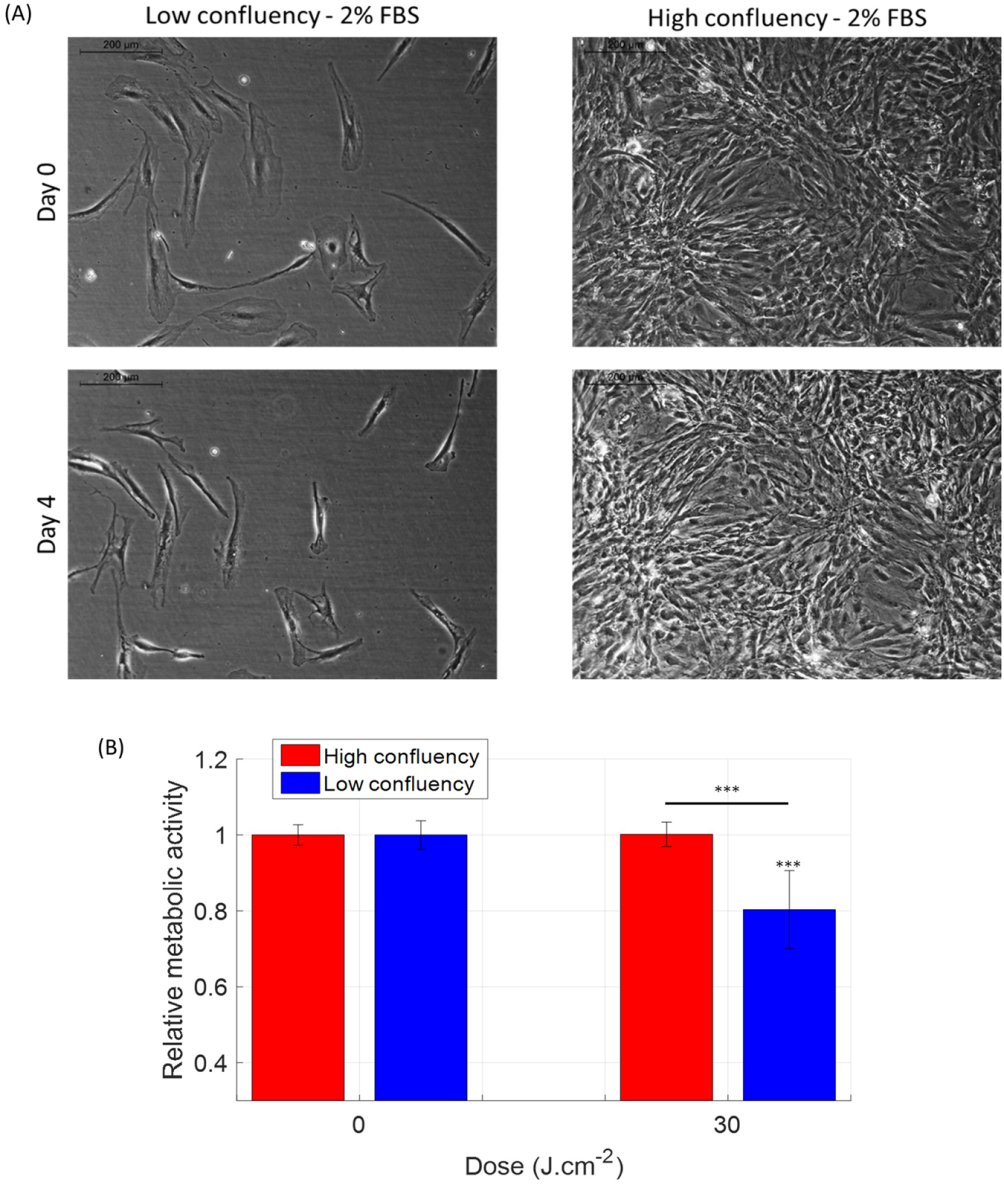
An increase in the cell confluency of human dermal fibroblasts reduces the inhibitory effect of blue light (450 nm) on their metabolic activity. (**A**) Phase-contrast images of human papillary fibroblasts on day 0 (8 days after seeding) at a low initial confluency (upper left) and high initial confluency (upper right), and after three light treatments on day 4 at a low initial confluency (lower left) and high initial confluency (lower right). Reticular fibroblasts were found to behave similarly (not shown). (**B**) Relative metabolic activity of human papillary fibroblasts after light treatment at 450 nm, 50 mW.cm^−2^ with 30 J.cm^−2^ in function of the confluency level. Statistical analysis was evaluated using student t-tests between the control group and the treated group (N = 3, 2 lineages and 3 replicates), with following thresholds *p < 0.05, **p < 0.01, ***p < 0.001. The control group has an average relative metabolic activity of 1. The significance of the interaction between the radiant exposure and the confluency level was much lower than 0.001.

To further investigate the effect of defined light parameters on DF metabolic activity, experiments were carried out at two different cell confluency levels: low (20%, 5,000 cells per 1.77 cm^2^ well area at seeding) and a very high (90%, 30,000 cells per 1.77 cm^2^ well area at seeding) (Fig. 4A). Remarkably, the same light parameter applied to what is typically assumed in the PBM literature as ‘identical’ cells, albeit at different confluencies, resulted in opposite effects (Fig. 4B). This was clearly different at two dose levels used, i.e., 2 and 30 J.cm^−2^ (only the higher dose is shown). It appears that DF at high confluencies (>90%) may be more ‘protected’ in response to 450 nm light, at least in context of metabolic activity.

### Impact of the treatment regimes and protocols on dermal fibroblast metabolic activity

**Figure 5:**
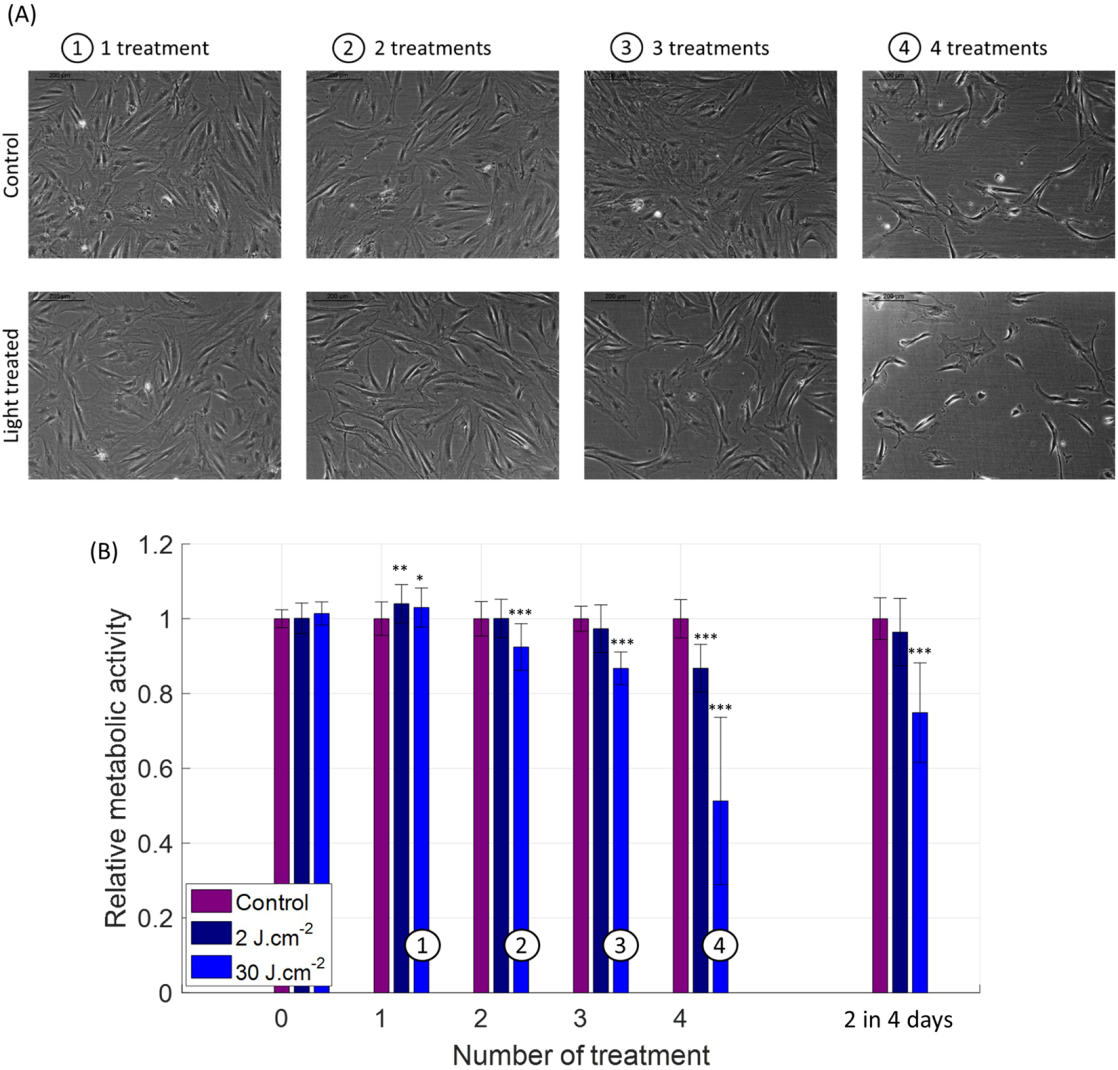
Response of human dermal fibroblasts to variable number of treatments with blue light (450 nm) occurring on consecutive days. (**A**) Phase-contrast images of human reticular fibroblasts 24 h after the last treatment, when 1, 2, 3 or 4 daily treatments (30 J.cm^−2^) were performed (lower line), together with the images of the control group (upper line). (**B**) Relative metabolic activity of human papillary and reticular fibroblasts (merged) after light treatment for 2 radiant exposures of 450 nm light (at a fixed 50 mW.cm^−2^ irradiance) and with variable number of treatments occurring on consecutive days. An irradiance of 50 mW.cm^−2^ was used. Statistical analysis was evaluated using student t-tests between the control group and treated group (N = 2, 2 lineages and 3 replicates), with following thresholds *p < 0.05, **p < 0.01, ***p < 0.001. The control group has an average relative metabolic activity of 1. The significance of the interaction between the radiant exposure and the number of iterative treatment was much lower than 0.001.

The choice of treatment regime, in particular, the number of consecutive treatments, impacts on the cell response to light, reflected in both cell metabolic activity and cell morphology. More specifically, a single exposure to 2 J.cm^−2^ and 30 J.cm^−2^ of 450 nm light resulted in an increase in metabolic activity, while 2, 3 or 4 consecutive treatments (Fig. 5B) reversed the effect, leading to decrease in cell metabolism.

Observed changes in metabolic activity were accompanied by morphological alterations (Fig. 5A), such as shrinkage of cells, which became more pronounced with an increase in the number of exposures. After 4 treatments, cells were significantly smaller than the corresponding control cells. This observed steady shrinking of cell volume could reflect the observed decrease in cell metabolic activity. A similar effect was also present in cells grown at low confluency (Fig. 4A).

Reducing light treatment frequency, from daily, every 24 hours, to every other day, every 48 hours, did not change the observed drop in metabolic activity or effect of cell morphology (Fig. 5B), suggesting that the initial exposure triggers a chain of molecular reactions lasting longer than 24 hours.

**Figure 6:**
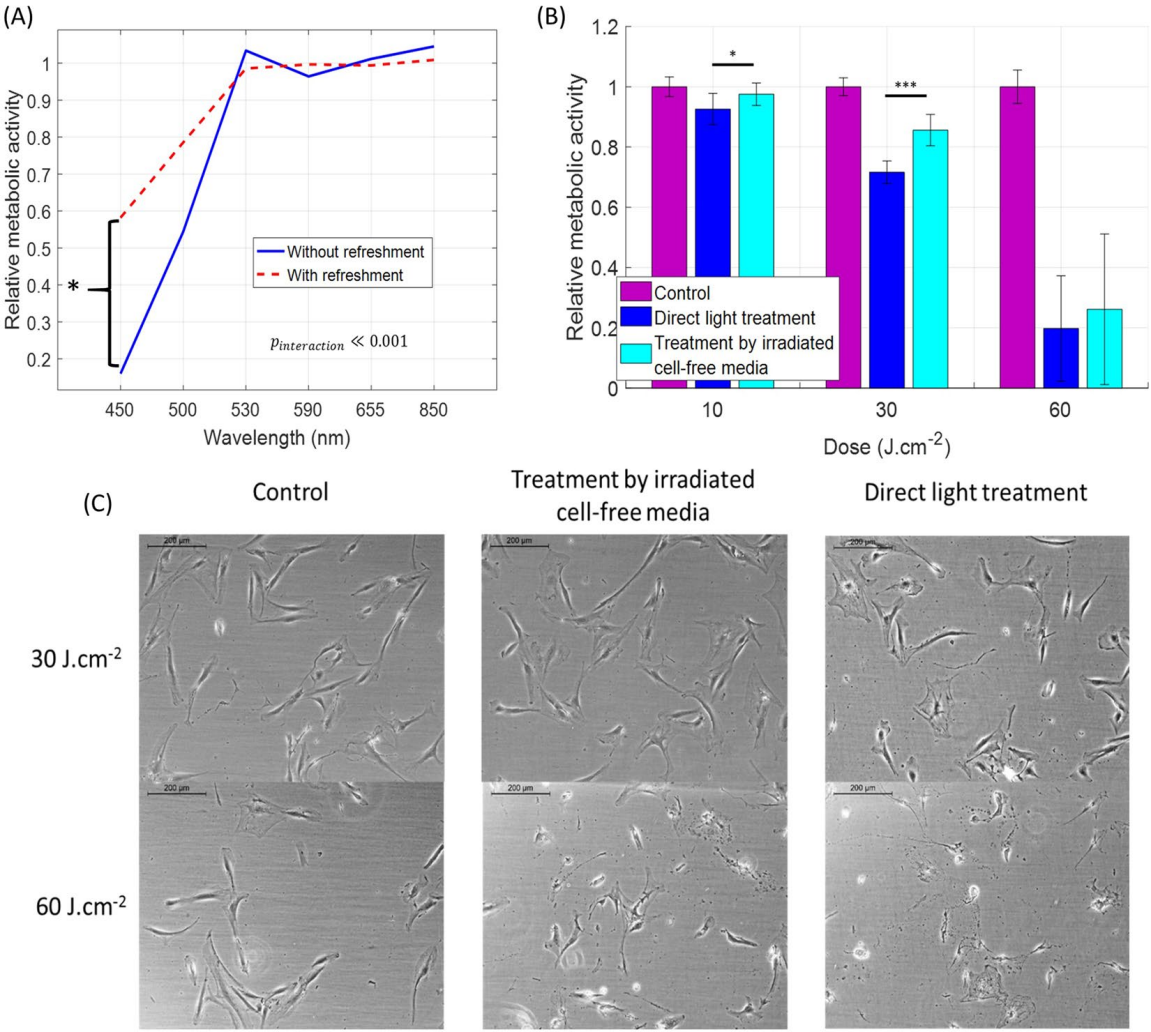
The inhibitory effect of blue light (450 nm) on the metabolic activity of human dermal fibroblasts is mediated by components present in the culture medium. (**A**) Relative metabolic activity of fibroblasts after light treatment as a function of the wavelength and the treatment regime (one or more consecutive irradiations) and protocol (direct light exposure or contact with pre-exposed culture medium). Metabolic activity after treatment was normalized to that of the control sample. Number of donors N = 3, number of technical replicates n = 3, data on papillary and reticular pools were merged together. The significance of the interaction between wavelength and treatment method was evaluated with ANOVA (p ≪ 0.001). (**B**) Relative metabolic activity of the fibroblasts after light treatment for two treatment protocols: direct light exposure (blue) and pre-exposure of DMEM alone followed by pouring on top of the cells (cyan). (**C**) Phase-contrast pictures of human reticular fibroblasts 24 h after the last treatment, in control group, treated group (30 J.cm^−2^ and 60 J.cm^−2^) and treated by irradiated cell-free media. An irradiance of 50 mW.cm^−2^ was used. Statistical analysis was evaluated using student t-tests between the control group and treated group (N = 3, reticular and 3 replicates), with following thresholds *p < 0.05, **p < 0.01, ***p < 0.001. The control group is has an average relative metabolic activity of 1.

Secondly, the treatment protocol used, in particular the culture medium components exerted a significant impact on the outcome of the light exposure to short visible wavelengths (Fig. 6A).

Previously, we have shown that exposure of DF to blue light of 450 nm (30 J.cm^−2^) in culture media (DMEM), without its subsequent refreshment, resulted in cytotoxic effects 24 hours after treatment. Here we observed a similar effect at the wavelength up to 530 nm, suggesting that a wide range of short visible wavelengths are absorbed by components of the cell culture media, leading to generation of molecules further impacting cell physiology.

We next assessed whether this effect was dependent on cell-free elements of the culture conditions, and found that DF exposure to cell-free media, irradiated using 30 J.cm^−2^ and 60 J.cm^−2^, resulted in reduced metabolic activity of the cells. The magnitude of this affect however, was lower than when cells in culture were directed irradiated (Fig. 6B), though fibroblast morphology was impaired only at 60 J.cm^−2^ (Fig. 6C).

### Impact of the oxygen level in cell culture medium on cellular response to light

Recent evidence suggests the existence of gradients of physiological oxygen levels throughout the different layers of human skin layers, where oxygen level in dermis (outside capillary loops) can be as low as 1–5%, compared to 20% atmospheric level^33, 34^. We therefore studied the effects of lowering oxygen concentration from 20% to 2% in our fibroblast cultures; i.e., to a physiologically-relevant concentration of oxygen in terms of DF interaction with visible light *in vivo*.

**Figure 7:**
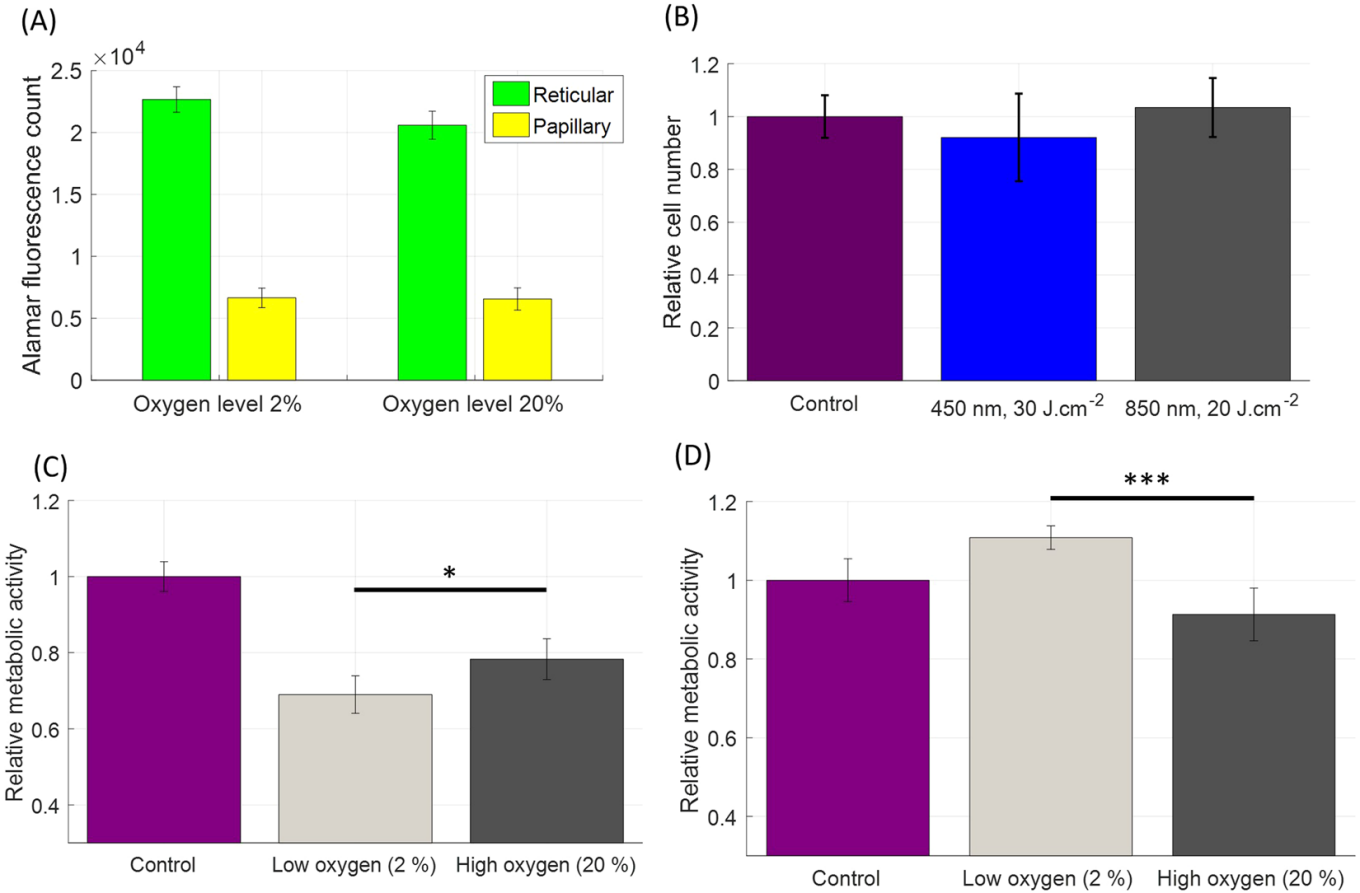
Impact of blue (450 nm) and NIR (850 nm) light treatment on the metabolic activity of human dermal fibroblasts when exposed to dermal tissue oxygen levels (approx. 2%). (**A**) Fluorescence counts (Alamar Blue) measured in reticular and papillary fibroblasts (facelift, female, 64 yo) in low (2%) and high (20%) oxygen levels. No strong change in Alamar fluorescence count is apparent. (**B**) Relative cell counts of human dermal fibroblasts (reticular and papillary) after light treatment in hypoxic conditions (2% oxygen). Cells were grown in 35-mm individual dishes and treated with light once a day for three consecutive days. Cell counting was performed 24 h after last treatment. Material originating from two different donors was included in the experiment (N = 2, reticular and papillary, 3 repeats, 12 counts per bar). (**C**) Relative metabolic activity of human papillary fibroblasts in response to 30 J.cm^−2^ at 450 nm in low and high environmental oxygen levels (N = 2, papillary and 3 replicates). (**D**) Relative metabolic activity of human reticular fibroblasts in response to 20 J.cm^−2^ at 850 nm in low and high environmental oxygen levels (N = 2, reticular and 3 replicates). Irradiance levels were 50 mW.cm^−2^ (450 nm) and 80 mW.cm^−2^ (850 nm).

The reduction from 20% to 2% oxygen concentration did not result in noticeable changes in the metabolic activity or morphology in control fibroblast (papillary and reticular) cultures, (Fig. 7A).

By contrast, lowering oxygen concentration resulted in dramatic differences in fibroblast response to light. In particular, blue light (450 nm) at 30 J.cm^−2^ resulted in even stronger inhibition of metabolic activity of papillary fibroblasts than when grown in 20% oxygen. Remarkably, NIR light (850 nm) at 20 J.cm^−2^ stimulated metabolic activity of human reticular fibroblasts at physiological levels of oxygen (i.e. 2%), the effect that was not observed at 20% oxygen level. These effects were not associated with any significant change on the relative cell number (Fig. 7B).

## Discussion and Conclusions

The goal of this study was to investigate the impact of optical parameters, cell culture protocols and treatment regimes on the metabolic activity of primary human dermal fibroblasts *in vitro* culture in response to visible and near-infrared radiation.

To achieve this we systematically studied the impact of wavelength, irradiance, dose, number of consecutive irradiations, serum- and oxygen concentration, cell confluency, medium refreshment and indirect treatment with irradiated medium alone in a design-of-experiment approach. The latter was specifically chosen in order to disentangle the effects caused by a direct interaction of photons of light with cells from the potential contribution of multiple ‘environmental’ factors.

We also aimed to explore whether the response of DF to identical optical parameters would change when cultured at conditions that more closely resemble those *in vivo*, i.e. low proliferation, cell confluency, serum and oxygen levels, as we felt that this is crucial when considering application of new technologies to *in vivo* clinical settings.

Several interesting and unexpected findings resulted from the approach, new to the world of photobiomodulation, which cause us to step back and re-assess how we interpret PBM data derived from *in vitro* studies.

First, all investigated parameters in this study, with the exception of irradiance, significantly impacted skin fibroblast metabolic responses to light.

Secondly, cell culture and treatment conditions (including confluency, serum- and environmental oxygen concentrations and treatment protocols) all significantly influence cellular responses to optical radiation, even when the identical wavelength, irradiance and dose were applied.

While medium-to-high confluency scenario is convention cell culture practice worldwide*, in vivo* DF are sparsely distributed throughout papillary and reticular dermal compartments. In an attempt to observe cells in a more natural situation, cultured at low confluency, we noticed that three consecutive daily treatments using short-wavelength visible light (450 nm at 30 J.cm^−2^) reduced their metabolic activity to a greater extent than when maintained at medium-to-high confluency.

Similarly, we observed that DF grown in lower FBS concentration 2%, though still higher than a fibroblast in a non-wounded skin environment would experience, coped less efficiently with induced stress, and were more inhibited by several consecutive exposures to 450 nm light at 30 J.cm^−2^. This response was clearly different from that of fibroblasts cultured at 10% FBS who were less inhibited and even protected from light-associated reactive oxygen species (ROS), as evidenced by normal metabolic activity and morphology. Similarly to low confluency, lower serum exposure may be more physiologically relevant.

The latter two findings may suggest that ‘non-physiological’ highly confluent cell cultures of DF in 10% FBS may be ‘more protected’ from potential stressors induced by light, such as ROS^35, 36^, than the cells *in vivo*. Two factors could potentially play a role here. First factor is a lower proliferative activity and thus lower number of mitotic cells (known to be more vulnerable to noxious factors^37, 38^ of highly confluent cell cultures of DF. Secondly, we also hypothesize that the higher the number of cells exposed to the light treatment event the stronger the antioxidant defense may be against any ROS created by irradiation of the cell medium constituents, as the latter was kept constant in all experiments regarding of cell number per well.

In addition to cell confluency and serum concentration, we assessed whether continuing to expose fibroblasts to their irradiated medium post-treatment negatively impacted on their viability. Specifically, we have shown that high radiant exposures of short visible wavelengths (450 and 500 nm, at 30 and 60 J.cm^−2^) suppressed fibroblast metabolic activity, and even resulted in some cytotoxicity when the medium was not refreshed after irradiation. Importantly, irradiation of cell-free culture medium that was then exposed to the cells resulted in an inhibitory effect, suggesting that ROS released after light absorption by riboflavin known to be present in the culture medium may have been at least in part responsible for this observed inhibitory effect. Previously it was shown that riboflavin mediated cytotoxic effects in human skin melanoma cells after irradiation by blue light (470 nm)^39^. However, it may not be completely artefactual, as it is also known that flavins are present in normal human skin dermis^40^ where they are likely to respond to clinical PBM modalities. However, we observed greater effects with the irradiated cell plus medium combination (than with the irradiated medium treatment alone), suggesting that there is a distinct cellular–derived component to the light response, indicating that photons of visible light can indeed directly interact with DF cells. Previously, interesting effects were observed in cells irradiated by ionizing radiation where oxidative stress was transferred from the irradiated cell to non-irradiated cells, via the release of components in the medium and even via the irradiated supernatant alone, which received a name ‘bystander effect’^41, 42^. Several signaling molecules mediated the transfer of oxidative stress. Small molecules, such as Ca2+ or peptides, were transmitted via gap junction intercellular communication to neighboring cells. Larger molecules, which could also be released by the irradiated cells, could propagate the damage and include lipid peroxide products and cytokines and reach neighboring cells by diffusion^43, 44^.

Next to cell culture conditions, what is less clear in PBM literature is if and how the experimental outcome depends on a number of consecutive treatments. Our observation is that the effect of shorter wavelengths (450 nm–590 nm) is accumulated over increasing number of consecutive daily and every other day treatments, suggests that the light can have prolonged effects on dermal fibroblasts, beyond cessation of irradiation, via the initiation of downstream cascade of cell signaling.

A key finding of our study was the observation that oxygen concentration markedly impacted on the response of dermal fibroblasts to both short visible- and to near-infrared light. Lowering oxygen to physiological level (i.e. 2%) resulted in fibroblast stimulation by near-infrared wavelengths: an observation not seen when cells were exposed to conventional cell culture oxygen levels (i.e. 20%), and so our protocol may allow researchers to appropriately investigate the photobiomodulation effects on skin and hair health. Indeed, researchers have shown the importance of environmental oxygen concentration at the genomic and proteomic levels in cells from various body locations. Physiological levels of oxygen in the body are organ-dependent and generally different from the standard oxygen concentration not only in human skin compartments but also in brain, lungs, liver and other body organs^45^. This therefore raises the need to distinguish between ‘normoxia’ and ‘physioxia’, and how this could impact PBM studies conducted using *in vitro* cell culture.

Skin temperature in itself could represent another condition to optimize in the pursuit of *in vivo* approximation. In the case of skin, the temperature is commonly assumed to be around 34 °C^45^. However, finding the right skin temperature might be complex for at least two reasons. First, there is evidence showing that skin temperature is highly variable within the body and dependent on the ambient temperature^46^. Second, related to light treatment *in vivo*, there will be thermal effects involved due to the strong optical absorption of visible and NIR radiations by skin chromophores (melanin, blood).

Similarly, our data suggests that *in vitro* protocols may be more physiological if irradiation is done with cells in culture medium rather than in a buffer such as PBS, given that *in vivo* (both steady state and during wounding/wound-healing) interstitial fluids and extracellular matrix will contain a complex mixture of essential minerals, ions, growth factors, and other nutrients and ligands’, which likely contribute to the total cellular response to light. However, the lack of *in vivo* ‘flow’ conditions in our static *in vitro* setup suggests that refreshment of medium after light treatment may be advisable. The downside being the loss of important autocrine cytokines and growth factors. In particular, we know that it may be important in the context of photobiomodulation, where latent growth factors have been shown to be activatable via infrared radiation^4^.

Interestingly, some of our study findings differ from those previously reported in the literature. First, our results did not show any strong effect of long visible and NIR radiation under environmental partial oxygen pressure. This observation is not surprising by itself, as the literature contains large discrepancies between the optical parameters and experimental outcomes used hitherto, impeding progress of PBM modality as previously reported^3^. More specifically, the majority of previous studies showed a measurable effect of red and NIR wavelengths *in vitro* and on *ex vivo* systems^4–6, 19, 47^. Other studies, however, found no effect of long visible and NIR wavelengths on skin cells^20, 48^. While not excluding the impact of cell culture conditions and oxygen levels on these, often opposite, experimental outcomes, we still hypothesize that human dermal fibroblasts, positioned deeper than epidermal melanocytes and keratinocytes, are more shielded from the blue part of the electromagnetic spectrum but are much more exposed to red and NIR components, and therefore could have adapted to them. Consequently one could suspect a stronger resistance of dermal fibroblasts to visible and NIR radiation, particularly in the long wavelength range, due to evolutionary reasons. The same reasoning may suggest that fibroblast originating from more deeply located tissues are more sensitive to light photons.

Second, we did not spot any irradiance effect *in vitro* despite some existing evidence in the literature both for *in vitro* and *in vivo* cases^49, 50^. However, irradiance effects have not been yet very clearly established *in vitro* in photobiomodulation. Although we might expect them *in vivo*, as light will be absorbed by skin chromophores (melanin, blood) and create bulk heat, it is less straightforward to expect irradiance effect *in vitro*. It might also have been that that our irradiance levels are not separated ‘well enough’ (only a factor 2 to 8 depending on the wavelength).

Third, we did not see any strong effect on the relative cell number after light treatment. However, the literature does contain evidence that short visible wavelengths exert anti-proliferative effects^20, 49^. The underlying reason of observed differences is that our biological model (low confluency, low serum and low oxygen) was tailored to resemble *in vivo* conditions of the dermal fibroblasts, where their proliferative activity is very low under non-wounded conditions. Combined with the replenishment of the media after light treatment, we barely can notice any proliferation for the duration of the experiments with light. Therefore, we were not expecting to be able to detect an effect on the relative cell number after treatment with light.

We conclude that *in vitro* experimental design factors including cell confluency, serum concentration, culture conditions like oxygen level, impact cell behavior^33, 51^ and so are critical to how we best define cell reactions to light i.e., either inhibitory, neutral or stimulatory, even when responding to light of the identical characteristics. Therefore, we recommend that for all *in vitro* and *ex vivo* investigations on photobiomodulation researchers should not only carefully select their experimental conditions as close to the physiological *in vivo* setting as possible but in any event report these together with their experimental findings. The implications of our study findings are that we recommend that confluency, serum- and environmental oxygen concentration should ideally be much lower than those currently used and reported worldwide in conventional dermal fibroblast culture protocols.

## Experimental Procedure and Methods

### Light-based devices

**Figure 8:**
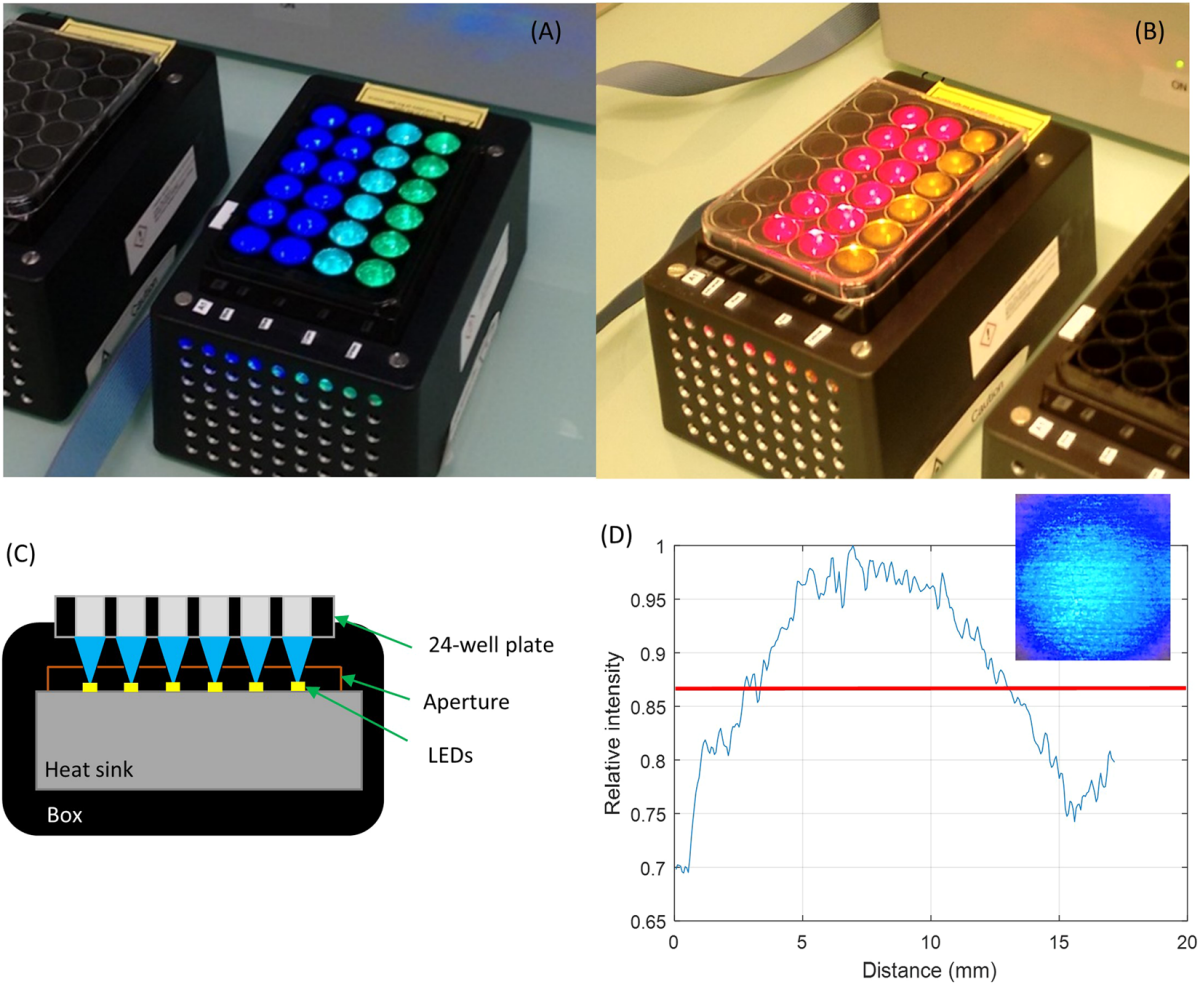
LED-based devices used in the experiments. (**A**) Photograph of sub-module 1 with the LEDs emitting 450, 500 and 530 nm wavelengths and (**B**) sub-module 2 with the LEDs emitting 590, 655 and 850 nm wavelengths. (**C**) Cross-section of the design a module, in the direction of a row of 6 wells of the 24-well plate (**D**) Typical profile of one single light beam in the axial x-y direction (at 450 nm) under one of the wells of the 24-well plate (the size of 1 well is 15 mm diameter) measured using a standard camera.

A proprietary LED-based device was designed to accommodate a single 24-well culture plate (Fig. 8) and had two sub-modules. Each module illuminated 4 rows of 6 wells. Each row of 6 wells was irradiated with the same wavelength. Rows of sub-module 1 were illuminated using 450, 500, 530 nm, while rows of sub-module 2 used 590, 655 and 850 nm light (Fig. 8A,B). Each sub-module had one row of wells without light treatment, which served as a control. Light leakage from well-to-well and thus light contamination was prevented by using blocking apertures between each individual LEDs (Fig. 8C) and by selecting black 24-well plate (Porvair Science, 303012).

The light beam under each well was created by a single LED (Lumileds LUXEON Rebel Color: Royal Blue 450 nm LXML-PR01–0500, Cyan 500 nm LXML-PE01–0070, Green 530 nm LXML-PM01–0090, Amber 590 nm LXML-PL01–0040 and Deep Red 655 nm LXM3-PD01–0300; OSRAM OSLON Black Series SFH4715S 850 nm) (Fig. 8C). The homogeneity of the illumination in x-y plane under each individual well, measured using a camera was within ±15% of the average and was found to be acceptable (Fig. 8D). The exposure time and irradiance settings were controlled via a computer interface. Optical power at the level of the target, i.e., at the bottom of the well-plate, was measured using Ophir Nova II power meter (Ophir Photonics) equipped with detector (PD300–3W). Bottom-illumination of the well-plate avoided unnecessary absorption of light by the support of the module and any potential increase in temperature, both of which can be a problem in case of top-based illumination.

### Primary human dermal fibroblast isolation and culture

Human dermal fibroblasts were isolated from fresh surplus residual human facial skin within 8 hours of surgery. Human skin was obtained after facelift surgery from healthy donors adhering to the Declaration of Helsinki principles under Human Tissue Transfer Agreement, between Philips Electronics Nederland B.V. acting through Philips Research and corresponding clinics, where clinics were responsible for having a full written informed consent from all patients. All experimental protocols were approved by the Philips Internal Committee of Biomedical Experiments (ICBE). Philips Electronics Nederland B.V., acting through Philips Research Eindhoven, confirms that it was its responsibility to ensure that approvals by appropriate local and/or national Ethics Committees and relevant authorizations were obtained whenever such approvals were required for the experiment(s).

Papillary and reticular fibroblasts lineages were extracted from superficial and deep dermis, respectively. Cells were initially cultured in DMEM supplemented with penicillin/streptomycin (1%), Glutamax (1%) and fetal bovine serum (FBS) (at 10%). The concentration of FBS was later very gradually reduced to 2% to quiesce fibroblast proliferation but not metabolic activity.

Pairs of reticular and papillary were donor sample-matched, unless otherwise indicated. The total number of donor used in the experiments was 4, the age of the donors was between 47 to 64 years old, the gender of the samples included both male and female and the passage was kept between passage 4 and 8 for all experiments.

### Light treatment protocols

The cells were treated daily over 3 consecutive days, during which the culture medium was refreshed after each light treatment, unless stated. Light treatment was conducted outside the culture incubator but never extended beyond a maximum of 20 minutes at room temperature. Any increase in temperature of the cell substratum due to the light treatment was assessed using a FLIR infrared camera (SC600). Thermal increase never exceeded 37 °C (i.e., body and culture incubator temperature) for all tested wavelengths and at all treatment time intervals. We observed very slight heating, originating from the light treatment (maximum of 2^o^C at 530 nm and 850 nm). This increase did not correlate with the effects that we observed on the fibroblast metabolic activity. Additionally, as treatment is done outside the incubator, the temperature of cells never exceeded the physiological range of skin temperature^46^. In a typical experiment the same 24-well plate contained both control and treated groups, as one row of the 24-well plate was always kept as control, i.e. not irradiated. This helped to control for any effect due to the treatment being performed outside the incubator. Indeed, both control and treated groups were always under the same ambient conditions. Light treatment was performed using transparent DMEM medium without phenol red (Sigma, D5921).

To assess any potential indirect impact of light on dermal fibroblasts via possible interactions with irradiated DMEM culture medium components, a cell-free DMEM medium was similarly irradiated using the same wavelengths and doses of light and immediately brought in contact with the test cells. The time of cell ‘exposure’ to light-irradiated cell-free medium was equal to that of treatment when light was applied directly on the cells.

### Measurement of dermal fibroblast metabolic activity

The metabolic activity of the dermal fibroblasts was assessed using the Alamar Blue® assay^52^ (Thermofisher, DAL1100). It was chosen for reported smallest variability across the different cell lineages, compared to MTT assay, often considered as the gold standard for determination of cell viability and proliferation^53^. MTT assay measures cell viability in terms of reductive activity as enzymatic conversion of the tetrazolium compound to water insoluble formazan crystals by dehydrogenases occurring in the mitochondria of living cells although reducing agents and enzymes located in other organelles such as the endoplasmic reticulum are also involved.

In contrast to MTT assay, in Alamar Blue® kit, the conversion of active component, resazurin, to fluorescent resorufin occurs mostly in the mitochondria and the quantity of resorufin generated can therefore be used as indicator of metabolic activity^54^.

On the day of assessment Alamar Blue® was added to fresh DMEM (10% in volume). The solution was then incubated in contact with the cells for 3 to 5 hours depending on the confluency of cells. The fluorescence generated was read using a plate reader FLUOstar Omega II (ex: 544 nm, em: 590 nm). The experimental outcome of the light-based treatments was expressed in terms of relative metabolic activity, defined as a ratio between the means of the treated groups and the control group.

### Assessment of viability and cell number

Automatic cell counting, Vi-CELL XR (Beckman Coulter Life Sciences), was used to assess the cell number. Cells were grown in 35-mm individual dishes and treated with light once a day for three consecutive days. The cells were counted 24 h after the last treatment. For each condition, the cells of 3 individual dishes were pooled together before counting. Cells counting was repeated three times. Material originating from two donors was included in the experiment (N = 2, reticular and papillary, 3 repeats, 12 counts per bar). Trypan blue is automatically added to assess viability as well.

### Assessment of cell morphology

Conventional phase-contrast light microscopy (Leica DMIL LED, obj. x10) was used daily to assess the morphological change of the dermal fibroblasts along treatment duration of three days.

### Design of experiment and statistical analysis

Previously, we described the statistical approach for a systematic assessment of the impact of optical and biological factors on the response of human dermal fibroblasts to light^24^. It is based on a factorial design of experiment, allowing identification of the factors having a significant impact on the variation of a variable under investigation, i.e., Alamar Blue® reading in case of this study. Such factors span a multidimensional space and are related to light treatment (i.e., optical variables) and the target and its environment (biological variables). Optical variables included: wavelength (nm), irradiance (mW.cm^−2^), radiant exposure or dose (J.cm^−2^), and biological variables included: serum concentration (% FBS), confluency (cells per units of surface), oxygen level (%) and treatment protocols (with or without replenishment of media after treatment, treatment with irradiated cell-free media).

Each factor was then varied among pre-defined levels, in our case – this was equal to 2, except for wavelength (where 6 discrete wavelengths were used). The level intervals were chosen to be large enough to show its impact, if present, on the variable of interest. The selected levels of the optical parameters are given in Table 1, while the levels of the biological factors are given in the Results section.

**Table 1:** Light parameters used for treatment of human dermal fibroblasts following a factorial design of experiment.

Parameter	Wavelength (nm)	Irradiance (mW.cm^−2^)	Radiant exposure (J.cm^−2^)	Parameter	Wavelength (nm)	Irradiance (mW.cm^−2^)	Radiant exposure (J.cm^−2^)
Blue-Low-Low	450	10	2	Yellow-Low-Low	590	3	2
Blue-Low-High	450	10	30	Yellow-Low-High	590	3	10
Blue-High-Low	450	50	2	Yellow-High-Low	590	7	2
Blue-High-High	450	50	30	Yellow-High-High	590	7	10
Cyan-Low-Low	500	10	2	Red-Low-Low	655	10	2
Cyan-Low-High	500	10	30	Red-Low-High	655	10	30
Cyan-High-Low	500	40	2	Red-High-Low	655	65	2
Cyan-High-High	500	40	30	Red-High-High	655	65	30
Green-Low-Low	530	10	2	IR-Low-Low	850	10	2
Green-Low-High	530	10	30	IR-Low-High	850	10	30
Green-High-Low	530	30	2	IR-High-Low	850	80	2
Green -High-High	530	30	30	IR-High-High	850	80	30

The pre-defined factors were then varied within the same experiment in a full factorial design mode. This allowed for a fair estimation of the effect of each factor independently, as well as any interaction between them. As a consequence, the number of experiments to be performed is equal to the number of levels to the power of the number of factors. This provided statistical power when assessing the effect of one single factor on the variation of our readout, as the variation of the response due to each of its level is weighted by the same combinations of all the other factors and levels. The resulting variation is therefore most probably due to the variation of this single factor itself.

Visually two representations for data were used, namely the main effects plot and the interaction plot. While the first shows the effects of each factor separately, the second one shows the interaction between the factors and how it impacts the variable of interest, i.e., metabolic activity. The numerical analysis is performed using ANOVA. When indicated, a Student’s t-test was used to compare two different conditions.

Our measurement method and setup were analyzed through a gage R&R approach. Operator, measuring instrument and method (plate reader, etc.), microplate type (black/white), illumination system and technical repeatability were tested. The results of the ANOVA analysis directly determined our selection of parameter and method to reduce the variability and reach satisfying level of reproducibility and repeatability^55^.
